# An enzymatically stable GIP/xenin hybrid peptide restores GIP sensitivity, enhances beta cell function and improves glucose homeostasis in high-fat-fed mice

**DOI:** 10.1007/s00125-016-4186-y

**Published:** 2016-12-21

**Authors:** Annie Hasib, Ming T. Ng, Victor A. Gault, Dawood Khan, Vadivel Parthsarathy, Peter R. Flatt, Nigel Irwin

**Affiliations:** SAAD Centre for Pharmacy and Diabetes, School of Biomedical Sciences, University of Ulster, Cromore Road, Coleraine, BT52 1SA Northern Ireland UK

**Keywords:** GIP, Glucose, Glucose homeostasis, Glucose-dependent insulinotropic polypeptide, High-fat feeding, Hybrid, Insulin secretion, Xenin

## Abstract

**Aims/hypothesis:**

Glucose-dependent insulinotropic polypeptide (GIP) and xenin, regulatory gut hormones secreted from enteroendocrine K cells, exert important effects on metabolism. In addition, xenin potentiates the biological actions of GIP. The present study assessed the actions and therapeutic utility of a (DAla^2^)GIP/xenin-8-Gln hybrid peptide, in comparison with the parent peptides (DAla^2^)GIP and xenin-8-Gln.

**Methods:**

Following confirmation of enzymatic stability, insulin secretory activity of (DAla^2^)GIP/xenin-8-Gln was assessed in BRIN-BD11 beta cells. Acute and persistent glucose-lowering and insulin-releasing effects were then examined in vivo. Finally, the metabolic benefits of twice daily injection of (DAla^2^)GIP/xenin-8-Gln was determined in high-fat-fed mice.

**Results:**

All peptides significantly (*p* < 0.05 to *p* < 0.001) enhanced in vitro insulin secretion from pancreatic clonal BRIN-BD11 cells, with xenin (and particularly GIP)-related signalling pathways, being important for this action. Administration of (DAla^2^)GIP or (DAla^2^)GIP/xenin-8-Gln in combination with glucose significantly (*p* < 0.05) lowered blood glucose and increased plasma insulin in mice, with a protracted response of up to 4 h. All treatments elicited appetite-suppressive effects (*p* < 0.05), particularly (DAla^2^)GIP/xenin-8-Gln and xenin-8-Gln at elevated doses of 250 nmol/kg. Twice-daily administration of (DAla^2^)GIP/xenin-8-Gln or (DAla^2^)GIP for 21 days to high-fat-fed mice returned circulating blood glucose to lean control levels. In addition, (DAla^2^)GIP/xenin-8-Gln treatment significantly (*p* < 0.05) reduced glycaemic levels during a 24 h glucose profile assessment. Neither of the treatment regimens had an effect on body weight, energy intake or circulating insulin concentrations. However, insulin sensitivity was significantly (*p* < 0.001) improved by both treatments. Interestingly, GIP-mediated glucose-lowering (*p* < 0.05) and insulin-releasing (*p* < 0.05 to *p* < 0.01) effects were substantially improved by (DAla^2^)GIP and (DAla^2^)GIP/xenin-8-Gln treatment. Pancreatic islet and beta cell area (*p* < 0.001), as well as pancreatic insulin content (*p* < 0.05), were augmented in (DAla^2^)GIP/xenin-8-Gln-treated mice, related to enhanced proliferation and decreased apoptosis of beta cells, whereas (DAla^2^)GIP evoked increases (*p* < 0.05 to *p* < 0.01) in islet number.

**Conclusions/interpretation:**

These studies highlight the clear potential of GIP/xenin hybrids for the treatment of type 2 diabetes.

**Electronic supplementary material:**

The online version of this article (doi:10.1007/s00125-016-4186-y) contains peer-reviewed but unedited supplementary material, which is available to authorised users.

## Introduction

A defect in the postprandial insulin-secretory incretin response, mediated by the gut hormones glucagon-like peptide-1 (GLP-1) and glucose-dependent insulinotropic peptide (GIP), is a specific pathophysiological characteristic of type 2 diabetes [1]. The main impairments are recognised as reduced postprandial GLP-1 secretion and defective GIP receptor signalling [1]. The inadequacy in the GLP-1 arm of the incretin effect can be easily overcome through administration of exogenous GLP-1, which significantly amplifies circulating concentrations [2, 3]. In contrast, pharmacological augmentation of circulating GIP levels fails to evoke an effective increase in insulin secretion in patients with type 2 diabetes [4]. As such, it seems unlikely that stand-alone GIP-based drugs would have therapeutic value for type 2 diabetes. Notwithstanding this, strategies to overcome defective GIP action in type 2 diabetes would be of considerable interest.

Near normalisation of blood glucose levels has been shown to restore the insulin-secretory effect of GIP in both animal models of type 2 diabetes [5] and in humans [6] with this condition, providing evidence that defective GIP receptor signalling is reversible. In addition, co-administration of GIP with a sulfonylurea restores pancreatic beta cell sensitivity to GIP [7], although this could be linked to uncoupling of incretin glucose dependency by sulfonylureas [8]. More encouraging, recent studies have highlighted the possibility that xenin, a hormone co-secreted with GIP from a subset of enteroendocrine K cells, could amplify the insulin-secretory response of GIP [9]. In agreement, observations from our laboratory and others confirm the GIP-potentiating effects of xenin under normal and type 2 diabetes conditions [10–13]. Furthermore, there is also evidence to suggest that xenin acts as a satiety hormone in animals [10, 14–17] and humans [18]. As such, therapeutic interventions that combine the biological actions of xenin and GIP, and potentially restore GIP action in type 2 diabetes, would have particularly exciting potential. There has been a recent upsurge in interest focused on generating designer hybrid peptides that can modulate multiple regulatory peptide hormone receptor pathways [19–22].

Successful generation of hybrid peptides has been achieved through fusion of the key bioactive amino acid sequences of the parent peptides [19–22]. This increases the therapeutic applicability of gut-hormone-based drugs by facilitating formulation and dosing with a single molecule, rather than co-injection of separate parent peptide forms. For xenin, the naturally occurring C-terminal fragment, known as xenin-8, retains biological activity at the level of the endocrine pancreas [13, 23]. Moreover, we have also shown that a stable analogue of xenin-8, namely xenin-8-Gln, is biologically active and has a spectrum of beneficial metabolic effects in vitro and in vivo [24]. For GIP, the first 14 N-terminal amino acid residues contain the bioactive domain important for insulin-secretory function [25, 26]. Based on this knowledge, we constructed a novel GIP/xenin hybrid peptide, (DAla^2^)GIP/xenin-8-Gln, by linking GIP(1-14) to xenin-8-Gln, retaining the regions of each peptide known to be important for biological activity (see electronic supplementary material [ESM] Table 1). Importantly, since GIP is a substrate for dipeptidyl peptidase-4 (DPP-4) [27], the hybrid peptide includes substitution of the naturally occurring alanine l isomer residue by a d isomer at position 2 [28, 29]. The results reveal that GIP/xenin hybrid molecules require further consideration as a treatment option for type 2 diabetes.

## Methods

### Peptide synthesis

All peptides (ESM Table 1) were purchased from GL Biochem (Shanghai, China; greater than 95% purity). Peptides were characterised in-house using HPLC and matrix-assisted laser desorption ionisation time-of-flight (MALDI-TOF) mass spectrometry (ESM Table 2), as described previously [29].

### Assessment of DPP-4 degradation

Peptide (20 μg) DPP-4 degradation profiles (5 μl purified DPP-4, 5 mU; Sigma-Aldrich, UK) were assessed as described previously [29]. Xenin-based peptides have been shown to be resistant to DPP-4 [10, 24].

### In vitro insulin secretion

BRIN-BD11 cells were used to assess the insulin-releasing activity of test peptides, as described previously [30]. This hybrid cell line (ECACC 10033003) was originally generated in-house by electrofusion of a primary culture of New England Deaconess Hospital (NEDH) rat pancreatic islets with RINm5F (a cell line derived from an NEDH rat insulinoma), and has been mycoplasma eradicated. For details of experimental conditions please see ESM Methods. Insulin was measured by radioimmunoassay [31]. We assessed the impact of GIP and neurotensin receptors on (DAla^2^)GIP/xenin-8-Gln-induced insulin secretion in BRIN-BD11 cells. Cells were incubated in 5.6 mmol/l glucose with (DAla^2^)GIP/xenin-8-Gln (10^−6^ mol/l) alone or in combination with the GIP receptor antagonist GIP(6-30)Cex-K^40^[Pal] [32] (where Pal is palmitate) or the neurotensin receptor antagonist SR142948A (Sigma, Poole, UK), and insulin secretion determined as described above. In addition, to determine mechanisms, intracellular Ca^2+^ was also measured in BRIN-BD11 cells following treatment with test peptides (10^−6^ mol/l), using a Flexstation scanning fluorometer (FLIPR Calcium 5 assay kit; Molecular Devices, Sunnyvale, CA, USA), as described previously [10].

### Animals

Acute and persistent dose-dependent animal studies were conducted in male albino NIH outbred Swiss mice (Envigo, Huntingdon, UK), that were derived from a nucleus colony obtained from the National Institutes of Health, Bethesda, Maryland. Longer-term experiments were conducted with male high-fat-fed NIH Swiss mice. For further details of mice age and maintenance please see ESM.

### Acute in vivo effects in lean mice

Specific information on acute feeding, glucose homeostasis and insulin-secretory studies is given in ESM Methods. To assess the impact of GIP and neurotensin receptor activation on (DAla^2^)GIP/xenin-8-Gln-induced glucose-lowering and insulin-secretory actions in mice, (DAla^2^)GIP/xenin-8-Gln was administered by i.p. injection (25 nmol/kg body weight) in combination with glucose (18 mmol/kg) in the absence and presence of GIP(6-30)Cex-K^40^[Pal] and SR142948A (both at 25 nmol/kg), and glucose and insulin were measured as described above. In a final series of experiments, test peptides (25 nmol/kg) or saline vehicle (0.9% wt/vol. NaCl) were injected 4 h before a glucose load (18 mmol/kg) in non-fasted mice and blood glucose and plasma insulin measured as above.

### Subchronic in vivo studies in high-fat-fed mice

Twice-daily (09:30 and 17:30 hours) i.p. injections of saline vehicle, (DAla^2^)GIP or (DAla^2^)GIP/xenin-8-Gln hybrid (both at 25 nmol/kg) were administered for 21 days in high-fat-fed mice. Energy intake and body weight were monitored daily and non-fasting blood glucose and plasma insulin concentrations were assessed at 3 day intervals. On day 21, a non-fasted 24 h glucose profile was conducted. At the end of the treatment period, i.p. glucose tolerance (18 mmol/kg), biological response to GIP (18 mmol/kg glucose in combination with native GIP [25 nmol/kg], i.p.) and insulin sensitivity (15 U/kg; i.p.) tests were performed. Terminal analysis included measurement of total body fat and lean mass by dual-energy X-ray absorptiometry (DEXA) scanning (Piximus Densitometer; Inside Outside Sales, Fitchburg, WI, USA) and extraction of pancreatic tissue for analysis as detailed below.

### Immunohistochemistry

Pancreas tissue was excised, divided longitudinally and either snap frozen for extraction of insulin using acid ethanol (5 ml/g), as described previously [22], or processed for immunohistochemical examination. Immunohistochemistry was performed as described previously [33]. For specific details, please see ESM Methods.

### Biochemical analysis

Blood samples were collected from the cut tip on the tail vein of conscious mice into chilled fluoride/heparin glucose micro-centrifuge tubes (Sarstedt, Numbrecht, Germany). Blood glucose was measured directly using a hand-held Ascencia Contour blood glucose meter (Bayer Healthcare, Newbury, UK). Plasma and pancreatic insulin was assayed by a modified dextran-coated charcoal radioimmunoassay [31].

### Statistical analysis

Statistical analysis was performed using GraphPad PRISM (La Jolla, CA, USA; version 5). For in vitro, islet histology and pancreatic insulin content studies, all samples were numbered and blinded. For acute and subchronic metabolic tests, there was no blinding. There were no inclusion and exclusion criteria applied. Results are expressed as means ± SEM and data were compared using repeated measures ANOVA followed by the Student–Newman–Keuls post hoc test. Groups of data were considered to be significantly different if *p* < 0.05.

## Results

### In vitro studies

**Fig. 1:**
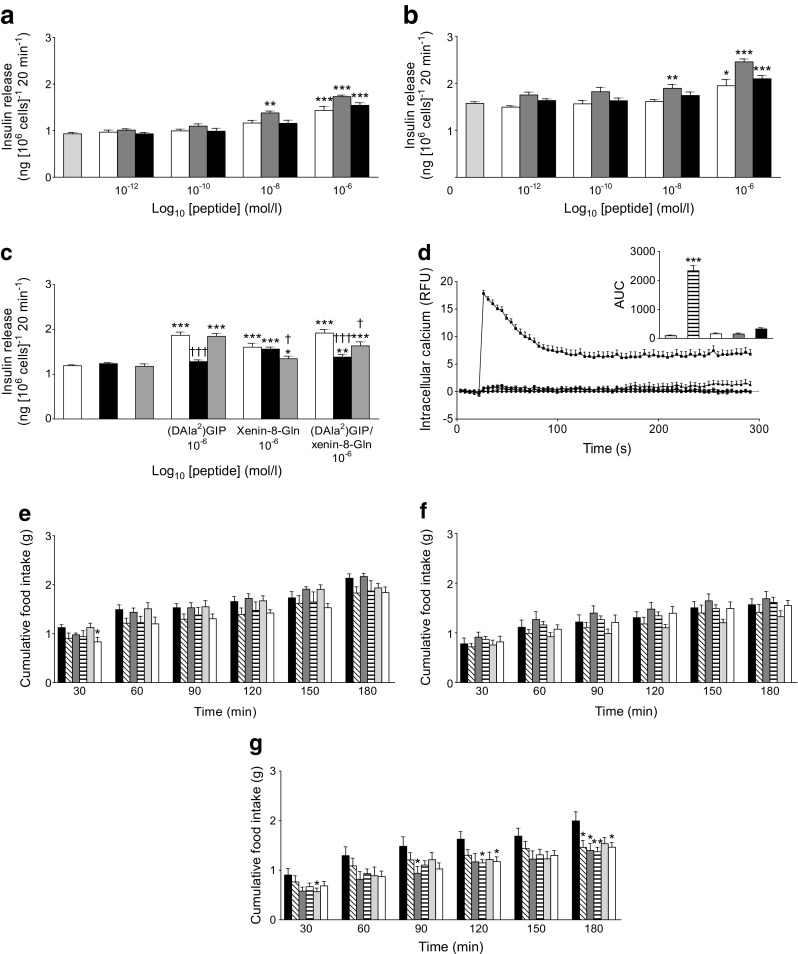
Effects of peptides on insulin release and intracellular Ca^2+^ concentrations in BRIN-BD11 cells and on cumulative food intake in lean control mice. (**a**, **b**) BRIN-BD11 cells were incubated (20 min) with test peptides in the presence of 5.6 mmol/l glucose (**a**) or 16.7 mmol/l glucose (**b**). Light-grey bars, glucose controls; white bars, xenin-8-Gln; dark-grey bars, (DAla^2^)GIP; black bars, (DAla^2^)GIP/xenin-8-Gln. (**c**) Effects of the GIP and neurotensin receptor antagonists, GIP(6-30)Cex-K^40^[Pal] and SR142948A, respectively, on (DAla^2^)GIP-, xenin-8-Gln- and (DAla^2^)GIP/xenin-8-Gln-mediated (20 min) insulin release in BRIN-BD11 cells. White bars, incubation in 5.6 mmol/l glucose alone; black bars, glucose plus GIP(6-30)Cex-K^40^[Pal] (10^−6^ mol/l); grey bars, glucose plus SR142948A (10^−6^ mol/l). (**d**) BRIN-BD11 cells were incubated with 5.6 mmol/l glucose in the presence of test peptides (10^−6^ mol/l) and intracellular Ca^2+^ concentrations were assessed over a 5 min period, with alanine (10 mmol/l) as positive control. AUC (0–300 s) data is shown in the inset. Black circles and light-grey bars, 5.6 mmol/l glucose control; black squares and striped bars, 10 mmol/l alanine; white circles and white bars, xenin-8-Gln; white squares and dark-grey bars, (DAla^2^)GIP; black triangles and black bars, (DAla^2^)GIP/xenin-8-Gln. (**e**–**g**) Cumulative food intake was measured after i.p. injection of peptides at 25 (**e**), 100 (**f**) and 250 nmol/kg (**g**) in overnight-fasted (18 h) lean control mice. Black bars, saline control; diagonally striped bars, xenin-8; dark-grey bars, xenin-25; horizontally striped bars, xenin-8-Gln; light-grey bars, (DAla^2^)GIP; white bars, (DAla^2^)GIP/xenin-8-Gln. Values represent means ± SEM (*n* = 8). *p < 0.05, **p < 0.01 and ***p < 0.001 compared with respective glucose (**a**–**d**) or saline controls (**e**–**g**); ^†^ *p* < 0.05 and ^†††^ *p* < 0.001 compared with incubations in the absence of GIP or neurotensin receptor antagonists, as appropriate

In contrast to native GIP, (DAla^2^)GIP/xenin-8-Gln and (DAla^2^)GIP remained fully intact when incubated in the presence of DPP-4 for up to 12 h (ESM Table 2). All peptides significantly (*p* < 0.05 to *p* < 0.001) stimulated insulin secretion from BRIN-BD11 cells at a concentration of 10^−6^ mol/l (Fig. 1a, b). Only (DAla^2^)GIP evoked a significant (*p* < 0.01 to *p* < 0.001) increase in insulin release at 10^−8^ mol/l when compared with respective glucose controls (Fig. 1a, b). As expected, the GIP receptor inhibitor GIP(6-30)Cex-K^40^[Pal] completely annulled (DAla^2^)GIP-mediated elevation of insulin secretion, while the neurotensin receptor inhibitor SR142948A had a similar, but less prominent, effect on xenin-8-Gln (Fig. 1c). As such, insulin concentrations were still significantly elevated (*p* < 0.05) following co-culture of xenin-8-Gln and SR142948A when compared with 5.6 mmol/l glucose control (Fig. 1c). The insulinotropic effect of (DAla^2^)GIP/xenin-8-Gln was completely inhibited (*p* < 0.001) by GIP(6-30)Cex-K^40^[Pal] and partially reversed (*p* < 0.05) by SR142948A (Fig. 1c). Neither, (DAla^2^)GIP, xenin-8-Gln nor (DAla^2^)GIP/xenin-8-Gln had any effect on intracellular Ca^2+^ concentrations (Fig. 1d).

### Acute in vivo food-intake studies

At 25 nmol/kg, (DAla^2^)GIP/xenin-8-Gln induced a significant (*p* < 0.05) reduction in food intake at 30 min post injection when compared with saline controls (Fig. 1e). None of the peptides had significant appetite-suppressive effects when administered at 100 nmol/kg (Fig. 1f). At a supraphysiological dose of 250 nmol/kg, all peptides except (DAla^2^)GIP induced significant (*p* < 0.05 to *p* < 0.01) reductions in food intake at 180 min post injection (Fig. 1g). In addition, at 250 nmol/kg, the appetite-suppressive effect of xenin was significantly (*p* < 0.05) superior to control mice at 90 min post injection, while xenin-8-Gln and (DAla^2^)GIP/xenin-8-Gln also evoked significant (*p* < 0.05) reductions in food intake at 120 min (Fig. 1g).

### Acute and persistent glucose-lowering and insulin-releasing effects in lean mice

**Fig. 2:**
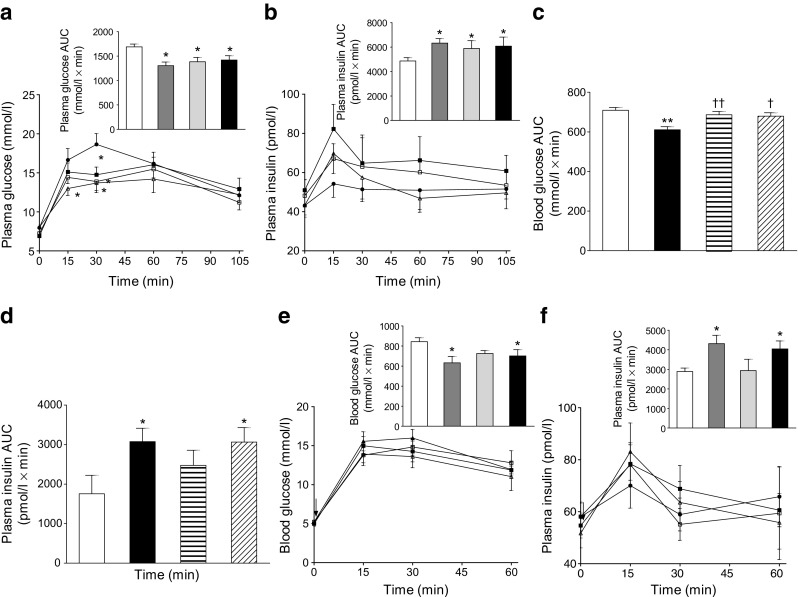
Acute and persistent glucose-lowering and insulin-releasing effects of (DAla^2^)GIP, xenin-8-Gln and (DAla^2^)GIP/xenin-8-Gln in lean control mice. (**a**, **b**) Blood glucose (**a**) and plasma insulin (**b**) concentrations were measured before and after i.p. injection of glucose alone (18 mmol/kg) or in combination with peptides (each at 25 nmol/kg) in non-fasted mice. AUCs are shown in insets. (**c**, **d**) Overall AUCs for blood glucose (**c**) and plasma insulin (**d**) were measured before and after i.p. injection of (DAla^2^)GIP/xenin-8-Gln. (25 nmol/kg) in combination with glucose (18 mmol/kg) in the absence or presence of GIP(6-30)Cex-K^40^[Pal] and SR142948A (both at 25 nmol/kg) in non-fasted mice. (**e**, **f**) Blood glucose (**e**) and plasma insulin (**f**) concentrations were measured following an i.p. glucose load (18 mmol/kg) in non-fasted mice injected with saline vehicle or peptides (each at 25 nmol/kg) 4 h previously. AUCs are shown in insets. Black circles and white bars, glucose alone (**a**–**d**) or saline control (**e**, **f**); white triangles and dark-grey bars, glucose (**a**, **b**) or saline (**e**, **f**) in combination with (DAla^2^)GIP; white squares and light-grey bars, glucose (**a**, **b**) or saline (**e**, **f**) in combination with xenin-8-Gln; black squares and black bars, glucose (**a**–**d**) or saline (**c**, **d**) in combination with (DAla^2^)GIP/xenin-8-Gln; horizontal striped bars, (DAla^2^)GIP/xenin-8-Gln in combination with GIP(6-30)Cex-K^40^[Pal]; diagonally striped bars, (DAla^2^)GIP/xenin-8-Gln in combination with SR142948A. Values represent means ± SEM for 7 or 8 mice. *p < 0.05 and **p < 0.01 compared with glucose alone (**a**, **b**) or saline control (**c**, **d**); ^†^ *p* < 0.05 and ^††^ *p* < 0.01 compared with (DAla^2^)GIP/xenin-8-Gln group

Administration of xenin-8-Gln, (DAla^2^)GIP or (DAla^2^)GIP/xenin-8-Gln concomitantly with glucose resulted in significantly (*p* < 0.05) lowered blood glucose values at 30 min post injection, culminating in significantly (*p* < 0.05) decreased overall AUC blood glucose values when compared with controls (Fig. 2a). (DAla^2^)GIP also induced a significant (*p* < 0.05) reduction in blood glucose levels 15 min post injection (Fig. 2a). Corresponding glucose-induced plasma insulin concentrations were not altered between groups in terms of individual values, although overall glucose-stimulated plasma insulin levels were significantly (*p* < 0.05) increased in all treatment groups compared with controls (Fig. 2b). The beneficial (*p* < 0.01) acute glucose-lowering effect of (DAla^2^)GIP/xenin-8-Gln was blocked by both GIP(6-30)Cex-K^40^[Pal] and SR142948A (Fig. 2c), whereas only GIP(6-30)Cex-K^40^[Pal] impaired (DAla^2^)GIP/xenin-8-Gln-induced insulinotropic actions (Fig. 2d). When administered 4 h prior to a glucose load, xenin-8-Gln was devoid of glucose-lowering or insulin-releasing effects (Fig. 2e, f). However, when administered 4 h previously, both (DAla^2^)GIP and (DAla^2^)GIP/xenin-8-Gln significantly reduced the overall AUC for blood glucose (*p* < 0.05) and increased plasma insulin AUC (*p* < 0.05) following a glucose challenge (Fig. 2e, f).

### Effects of twice-daily administration of (DAla^2^)GIP and (DAla^2^)GIP/xenin-8-Gln on energy intake, body weight, non-fasted blood glucose and plasma insulin in high-fat-fed mice

**Fig. 3:**
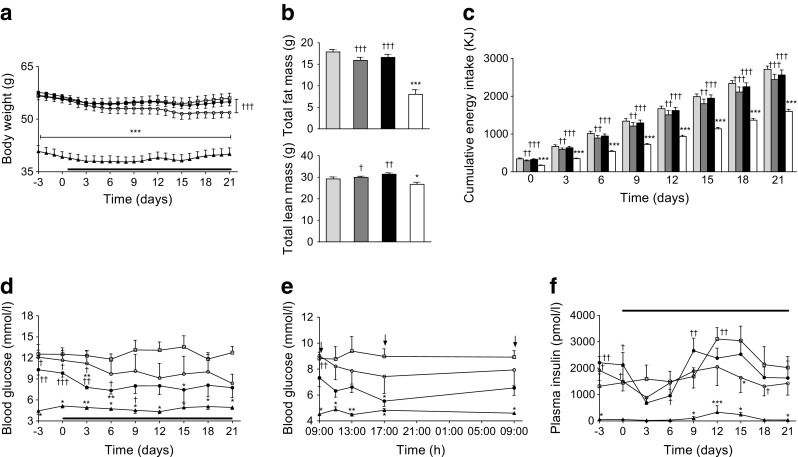
Effects of twice-daily administration of (DAla^2^)GIP and (DAla^2^)GIP/xenin-8-Gln on body weight, body composition, cumulative energy intake, non-fasted blood glucose, 24 h blood glucose profile and non-fasted plasma insulin in high-fat-fed mice. (**a**, **c**, **d**, **f**) Variables were measured for 3 days before and 21 days during (indicated by black horizontal line) twice-daily treatment with saline vehicle, (DAla^2^)GIP or (DAla^2^)GIP/xenin-8-Gln hybrid (each at 25 nmol/kg). (**b**, **e**) Total fat and lean mass (**b**) and 24 h glucose profile (**e**) were assessed on day 21. Arrows indicate timing of normal twice-daily injections. White squares and light-grey bars, saline control; white circles and dark-grey bars, (DAla^2^)GIP; black circles and black bars, (DAla^2^)GIP/xenin-8-Gln; black triangles and white bars, lean control. Values represent means ± SEM for 6–8 mice. *p < 0.05, **p < 0.01 and ***p < 0.001 compared with high-fat-fed controls. ^†^ *p* < 0.05, ^††^ *p* < 0.01 and ^†††^ *p* < 0.001 compared with lean controls

Twice-daily administration of (DAla^2^)GIP or (DAla^2^)GIP/xenin-8-Gln for 21 days to high-fat-fed mice had no significant effect on cumulative energy intake or body weight compared with high-fat-fed controls (Fig. 3a, c). In addition, total body fat and lean mass were also unaltered after the 21 day regimen (Fig. 3b). However, all high-fat fed mice exhibited significantly increased (*p* < 0.05 to *p* < 0.001) body weight and cumulative energy intake compared with lean controls (Fig. 3a, c). Non-fasting blood glucose levels progressively declined in (DAla^2^)GIP/xenin-8-Gln-treated high-fat fed mice over the 21 days, being significantly (*p* < 0.05 to *p* < 0.01) reduced on observation days 3, 6 and 15 compared with levels in control high-fat mice (Fig. 3d). Both (DAla^2^)GIP- and (DAla^2^)GIP/xenin-8-Gln-treated mice had glucose levels that were not significantly different from lean controls from day 12 onwards (Fig. 3d). Analysis of non-fasting 24 h glucose profile on day 21 revealed that (DAla^2^)GIP/xenin-8-Gln treated mice had similar blood glucose levels to lean controls at each observation point and significantly (*p* < 0.05) reduced levels compared with high-fat-fed controls at 17:00 hours (Fig. 3e). Blood glucose levels in (DAla^2^)GIP-treated mice were also reduced, but not to the same extent as in (DAla^2^)GIP/xenin-8-Gln mice (Fig. 3e). Circulating plasma insulin levels were generally elevated in all high-fat-fed mice compared with lean controls (Fig. 3f). Mice treated twice daily with either (DAla^2^)GIP or (DAla^2^)GIP/xenin-8-Gln did have reduced plasma insulin levels compared with high-fat-fed controls on day 21 but the difference did not reach statistical significance (Fig. 3f).

### Effects of twice-daily administration of (DAla^2^)GIP and (DAla^2^)GIP/xenin-8-Gln on glucose tolerance and metabolic response to GIP in high-fat-fed mice

Treatment with (DAla^2^)GIP or (DAla^2^)GIP/xenin-8-Gln for 21 days reduced overall AUC for blood glucose levels of high-fat-fed mice following a glucose load but the difference failed to reach statistical significance (Table 1). Similarly, glucose-stimulated plasma insulin AUCs were reduced non-significantly by (DAla^2^)GIP or (DAla^2^)GIP/xenin-8-Gln treatment compared with high-fat controls (Table 1). All high-fat-fed mice had a significantly (*p* < 0.05 to *p* < 0.001) increased glycaemic excursion and overall insulin-secretory response compared with lean controls (Table 1). As illustrated in Table 1, (DAla^2^)GIP and (DAla^2^)GIP/xenin-8-Gln treatment significantly (*p* < 0.05) improved the glucose-lowering and insulin-releasing actions of native GIP; overall blood glucose AUC values were significantly (*p* < 0.05) decreased by 36% and 30%, respectively, compared with saline-treated control. Moreover, AUCs for glycaemic values were not different when compared with lean controls in response to administration of GIP in combination with glucose (Table 1). Corresponding GIP-induced elevations of plasma insulin concentrations were also significantly (*p* < 0.05 to *p* < 0.01) augmented in (DAla^2^)GIP- and (DAla^2^)GIP/xenin-8-Gln-treated high-fat-fed mice compared with high-fat-fed controls (Table 1).

**Table 1:** Effects of twice-daily administration of (DAla^2^)GIP and (DAla^2^)GIP/xenin-8-Gln on glucose tolerance, glucose-stimulated insulin and GIP-mediated glucose-lowering and insulin-secretory actions in high-fat-fed mice

Treatment group	Glucose tolerance test		Metabolic response to GIP	
	Blood glucose AUC (mmol/l × min)	Plasma insulin AUC (pmol/l × min)	Blood glucose AUC (mmol/l × min)	Plasma insulin AUC (pmol/l × min)
High-fat saline control	2062.0 ± 121.2	16,377 ± 3063	16,18.9 ± 148.1	9770 ± 2024
(DAla^2^)GIP	1714.7 ± 138.6^†^	11,253 ± 1590^†^	1029.5 ± 138.0*	20,308 ± 3805**^,†††^
(DAla^2^)GIP/xenin-8-Gln	1757.3 ± 154.6^†^	8423 ± 1121^†^	1130.1 ± 143.9*	17,741 ± 4016*^,††^
Lean control	1080.5 ± 85.7***	4237 ± 232.6*	787.4 ± 54.2**	3611 ± 411.9

Values represent means ± SEM for 6–8 mice. AUC values (0–105 min) for blood glucose and plasma insulin are shown

Tests were conducted after twice-daily treatment with saline vehicle, (DAla^2^)GIP or (DAla^2^)GIP/xenin-8-Gln (each at 25 nmol/kg) for 21 days. For glucose tolerance test, blood glucose and plasma insulin concentrations were measured before and 15, 30, 60 and 105 min after i.p injection of glucose (18 mmol/kg). For metabolic response to GIP, blood glucose and plasma insulin concentrations were measured before and 15, 30, 60 and 105 min after i.p injection of glucose (18 mmol/kg) in combination with GIP (25 nmol/kg). All studies were conducted in mice fasted for 18 h

*p < 0.05, **p < 0.01 and ***p < 0.001 compared with high-fat controls; ^†^ *p* < 0.05, ^††^ *p* < 0.01 and ^†††^ *p* < 0.001 compared with lean controls

### Effects of twice-daily administration of (DAla^2^)GIP and (DAla^2^)GIP/xenin-8-Gln on insulin sensitivity and pancreatic insulin content in high-fat fed mice

**Fig. 4:**
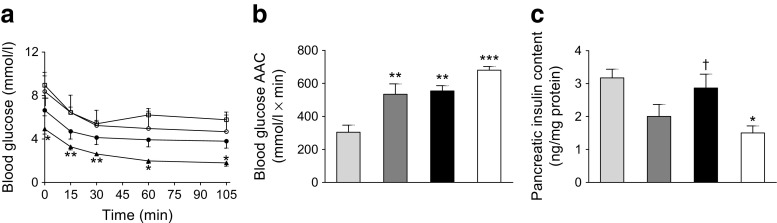
Effects of twice-daily administration of (DAla^2^)GIP and (DAla^2^)GIP/xenin-8-Gln on insulin sensitivity and pancreatic insulin content in high-fat-fed mice. Insulin (15 U/kg) was given by i.p. injection (*t* = 0) in non-fasted mice following 21 days treatment with saline vehicle, (DAla^2^)GIP or (DAla^2^)GIP/xenin-8-Gln hybrid (each at 25 nmol/kg). Blood glucose was measured (**a**), area above the curve (AAC) was calculated using identical baseline subtraction for each group (**b**) and pancreatic insulin content was measured by RIA (**c**). White squares and light-grey bars, saline control; white circles and dark-grey bars, (DAla^2^)GIP; black circles and black bars, (DAla^2^)GIP/xenin-8-Gln; black triangle and white bars, lean control. Values represent means ± SEM for 6–8 mice. *p < 0.05, **p < 0.01 and ***p < 0.001 compared with high-fat controls; ^†^ *p* < 0.05 compared with lean controls

Individual blood glucose levels were reduced, albeit non-significantly, in (DAla^2^)GIP- and (DAla^2^)GIP/xenin-8-Gln-treated high-fat-fed mice following administration of exogenous insulin (Fig. 4a). However, the overall glucose-lowering effect of insulin was significantly (*p* < 0.001) improved in (DAla^2^)GIP- and (DAla^2^)GIP/xenin-8-Gln-treated mice compared with control high-fat mice (Fig. 4b). The pancreatic insulin content of (DAla^2^)GIP/xenin-8-Gln-treated mice was augmented (*p* < 0.05) compared with that of lean controls but was not significantly different from that of high-fat controls (Fig. 4c). In contrast, the pancreatic insulin content of (DAla^2^)GIP-treated mice was similar to that of lean control mice (Fig. 4c).

### Effects of twice-daily administration of (DAla^2^)GIP and (DAla^2^)GIP/xenin-8-Gln on pancreatic islet histology in high-fat-fed mice

**Fig. 5:**
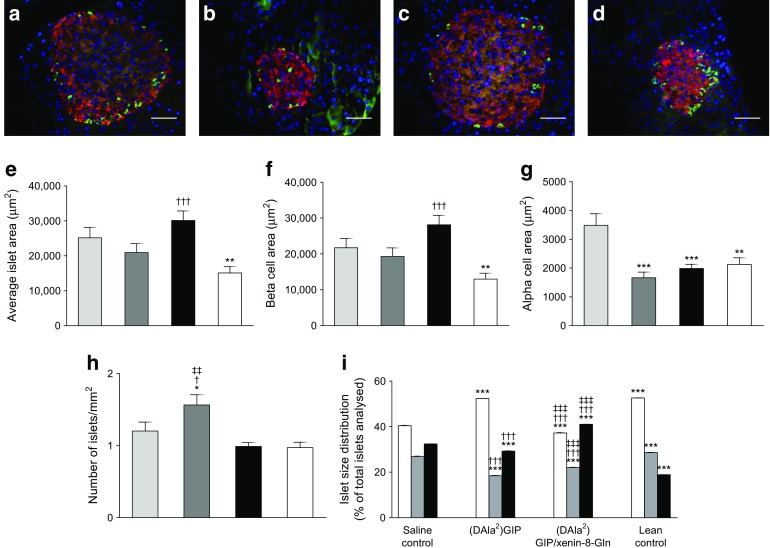
Effects of twice-daily administration of (DAla^2^)GIP and (DAla^2^)GIP/xenin-8-Gln on pancreatic histology. (**a**–**d**) Representative images of islets showing insulin (red) and glucagon (green) immunoreactivity from pancreatic tissues extracted from high-fat-fed saline control mice (**a**), (DAla^2^)GIP- (**b**) and (DAla^2^)GIP/xenin-8-Gln-treated high-fat-fed mice (**c**) and lean control mice (**d**). (**e**–**i**) Variables were assessed using Cell^F^ image analysis software after 21 days of twice-daily i.p injections of saline vehicle or peptides (each at 25 nmol/kg) in high-fat-fed mice. Light-grey bars, saline control; dark-grey bars, (DAla^2^)GIP; black bars, (DAla^2^)GIP/xenin-8-Gln; white bars, lean control (**e**–**h**). White bars, small islets (<10,000 μm^2^); grey bars, medium islets (10,000–25,000 μm^2^); black bars, large islets (>25,000 μm^2^) (**i**). Values are means ± SEM of 6–8 mice. Scale bar, 50 μm. *p < 0.05, **p < 0.01 and ***p < 0.001 compared with high-fat-fed controls. ^†^ *p* < 0.05 and ^†††^ *p* < 0.001 compared with lean controls; ^‡‡^ *p* < 0.01 and ^‡‡‡^ *p* < 0.001 compared with the (DAla^2^)GIP group

**Fig. 6:**
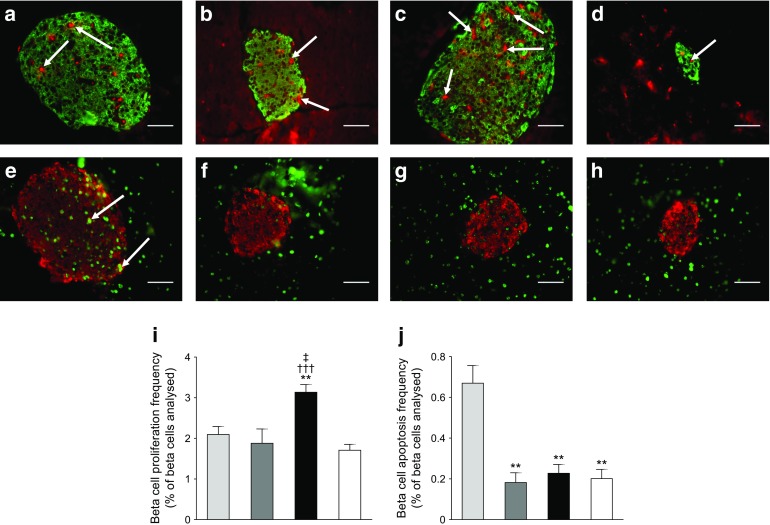
Effects of twice-daily administration of (DAla^2^)GIP and (DAla^2^)GIP/xenin-8-Gln on beta cell proliferation and apoptosis. (**a**–**d**) Representative images showing insulin (green) and Ki67 (red, indicated by arrows) in islets from high-fat-fed saline control mice (**a**), (DAla^2)^GIP- (**b**) and (DAla^2^)GIP/xenin-8-Gln-treated high-fat-fed mice (**c**) and lean control mice (**d**). (**e**–**h**) Representative images showing insulin (red) and TUNEL (green, indicated by arrows) immunoreactivity in islets from high-fat-fed saline control mice (**e**), (DAla^2)^GIP- (**f**) and (DAla^2^)GIP/xenin-8-Gln-treated high-fat-fed mice (**g**) and lean control mice (**h**). (**i**, **j**) Quantification of beta cell proliferation (**i**) and apoptosis frequency (**j**) expressed as a percentage of beta cells analysed. Light-grey bars, saline control; dark-grey bars, (DAla^2^)GIP; black bars, (DAla^2^)GIP/xenin-8-Gln; white bars, lean control. Approximately 5000 beta cells per experimental group were counted. Values are means ± SEM of 6–8 mice. Scale bar, 50 μm. **p < 0.01 compared with high-fat-fed controls; ^†††^ *p* < 0.001 compared with lean controls; ^‡^ *p* < 0.05 compared with (DAla^2^)GIP group

Representative images of pancreatic islets from each mouse group are shown in Fig. 5a–d. High-fat-fed mice had significantly (*p* < 0.01) increased islet area compared with lean controls (Fig. 5e), giving rise to significant (*p* < 0.01) increases in both beta and alpha cell areas (Fig. 5f, g). Treatment with (DAla^2^)GIP had no significant effect on overall pancreatic islet area or beta cell area but resulted in decreased (*p* < 0.001) alpha cell area compared with high-fat-fed controls (Fig. 5e–g). Similarly, 21 days of twice-daily treatment with (DAla^2^)GIP/xenin-8-Gln significantly (*p* < 0.001) reduced pancreatic alpha cell area compared with high-fat-fed controls (Fig. 5g). It also increased (*p* < 0.001) pancreatic islet and beta cell area compared with lean controls (Fig. 5e, f). The number of islets per mm^2^ was significantly (*p* < 0.05 to *p* < 0.01) augmented in (DAla^2^)GIP-treated mice compared with all other groups (Fig. 5h). This effect appeared to be related to an increase in number of small-sized islets (Fig. 5i). Thus, high-fat feeding significantly (*p* < 0.001) decreased the number of small islets and increased the number of large islets, compared with lean controls (Fig. 5i). (DAla^2^)GIP/xenin-8-Gln-treated high-fat-fed mice also had a decreased (*p* < 0.001) number of small- and medium-sized islets, and fewer (*p* < 0.001) large islets, when compared with lean controls (Fig. 5i). Representative images of Ki67 and TUNEL immunostained pancreatic islets from each group of mice are shown in Fig. 6a–h. Proliferation and apoptosis rates of pancreatic islet cells appeared to be relatively high in this strain of mouse. The rate of beta cell proliferation in (DAla^2^)GIP/xenin-8-Gln-treated high-fat-fed mice was significantly (*p* < 0.05 to *p* < 0.001) increased when compared with the rate in all other groups (Fig. 6i). In addition, the frequency of beta cell apoptosis was significantly (*p* < 0.01) decreased in (DAla^2^)GIP- and (DAla^2^)GIP/xenin-8-Gln-treated high-fat-fed mice compared with high-fat-fed controls, and was similar to levels in lean control mice (Fig. 6j).

## Discussion

Despite encouraging preclinical data and the success of DPP-4 inhibitors [34, 35], progression of enzyme-resistant GIP-based drugs to the type 2 diabetes clinic is lacking. Therefore, in the present study we have evaluated the biological actions and therapeutic applicability of a novel GIP/xenin hybrid peptide, namely (DAla^2^)GIP/xenin-8-Gln.

In harmony with previous studies [28, 29], (DAla^2^)GIP and the hybrid peptide (DAla^2^)GIP/xenin-8-Gln were completely resistant to degradation by DPP-4, indicating that the N-terminal d-Ala^2^ modification employed effectively masked the DPP-4 binding site. As expected, the parent peptides, (DAla^2^)GIP and xenin-8-Gln, stimulated insulin release from clonal pancreatic BRIN-BD11 beta cells at physiological and elevated glucose concentrations [24, 29]. To determine the influence of both GIP and xenin pathways on induction of bioactivity by (DAla^2^)GIP/xenin-8-Gln, we utilised the specific GIP receptor antagonist GIP(6-30)Cex-K^40^[Pal] [32] and the commercially available neurotensin receptor antagonist, SR142948A. Reassuringly, (DAla^2^)GIP/xenin-8-Gln was equally effective in vitro as an insulin secretagogue as either of the parent peptides. Its insulinotropic effects appeared to be more dependent on activation of GIP receptor-related cell signalling pathways, when compared with xenin. In full agreement, the acute in vivo insulinotropic actions of (DAla^2^)GIP/xenin-8-Gln were also chiefly dependent upon activation of GIP receptor pathways. Interestingly, the acute glucose-lowering actions of (DAla^2^)GIP/xenin-8-Gln appeared to be associated with activation of both GIP and xenin pathways, suggesting possible insulin-independent glucose-lowering effects of xenin [12]. However, the main effect of the xenin component of (DAla^2^)GIP/xenin-8-Gln likely relates to enhancement of GIP-mediated biological actions, as demonstrated here and previously [10, 11]. Full clarification of the mechanism of (DAla^2^)GIP/xenin-8-Gln-induced insulin release is necessary, but similar (DAla^2^)GIP/xenin-8-Gln-induced acute insulin-secretory effects were also noted in lean control mice, confirming biological efficacy. Our previous studies using clonal beta cells have shown that (DAla^2^)GIP activates adenylate cyclase pathways, whereas xenin-based peptides likely operate through the action of phospholipase C [10]. Further to this, we have shown that neither (DAla^2^)GIP/xenin-8-Gln nor its parent peptides significantly affected intracellular Ca^2+^ levels in BRIN-BD11 beta cells.

In accordance with preserved bioactivity of the hybrid peptide, we observed satiety effects of (DAla^2^)GIP/xenin-8-Gln even at the lowest dose examined and especially at elevated doses. This is in agreement with previous findings suggesting that elevated doses of xenin are required to impart significant appetite-suppressive activity, whereas lower doses have beneficial metabolic effects [10, 13]. The persistence of the metabolic actions of (DAla^2^)GIP/xenin-8-Gln was then assessed. Even 4 h after a single injection of (DAla^2^)GIP/xenin-8-Gln or (DAla^2^)GIP, glucose-lowering and insulin-releasing actions were clearly evident. This presumably relates to enhanced enzymatic stability of (DAla^2^) modified GIP-forms and corresponds well with the findings of other studies [28, 29]. Development of a specific assay to directly measure (DAla^2^)GIP/xenin-8-Gln in plasma would be useful to determine the pharmacokinetic profile of the hybrid peptide. Moreover, methodology such as peptide acylation or PEGylation may help to extend the biological half-life of (DAla^2^)GIP/xenin-8-Gln [29, 36]. Notably, the biological effects of (DAla^2^)GIP/xenin-8-Gln were equal, or indeed enhanced, when compared with either parent peptide administered at the same dose. This indicates that the hybrid peptide still fully retained the ability to activate GIP- and xenin-related cellular signalling pathways involved in glucose homeostasis, insulin secretion and appetite suppression [13, 29]. Taken together, these data provided a strong basis for the subsequent 21 day twice-daily injection regimen conducted in high-fat-fed mice.

Chronic treatment of high-fat-fed mice with (DAla^2^)GIP resulted in sustained and significant reductions of circulating blood glucose levels, both in terms of individual observation points and during assessment of a 24 h blood glucose profile [28, 29]. Crucially, (DAla^2^)GIP/xenin-8-Gln had equal or improved beneficial effects on glycaemic status in high-fat mice. Indeed, reductions in glucose levels were much more apparent in mice treated with (DAla^2^)GIP/xenin-8-Gln during assessment of the more-in-depth 24 h blood glucose profile. Interestingly, significantly greater reductions in insulin-induced blood glucose levels were observed in (DAla^2^)GIP/xenin-8-Gln- and (DAla^2^)GIP-treated mice. This clearly points towards beneficial effects of both treatments that are additional to effects on pancreatic beta cell function. This improvement of insulin action was not a consequence of reduced adipose tissue mass. Thus, it presumably reflects alleviation of glucotoxicity, or the actions of GIP to improve insulin resistance [29, 37]. However, further investigations, including euglycaemic–hyperinsulinaemic clamps, glucose uptake and gene and protein expression studies, are required to fully address the molecular mechanisms underpinning this benefit. Somewhat surprisingly, at the dose tested, (DAla^2^)GIP/xenin-8-Gln had no effect on energy intake, although similar observations have been reported previously with xenin-based drugs [38]. This limited satiety effect presumably reflects the requirement of supraphysiological doses of xenin to impart appetite-suppressive effects [10]. However, it could also be related to factors such as the palatability of the high-fat diet, the age and strain of mice employed or the duration of the study.

As expected, a key component of the beneficial action of (DAla^2^)GIP/xenin-8-Gln concerned the stimulation of beta cell function [13, 29]. Circulating and glucose-induced plasma insulin concentrations were significantly elevated compared with those in lean control mice, despite non-fasting glucose levels being similar. Moreover, the pancreatic insulin content was persistently higher in (DAla^2^)GIP/xenin-8-Gln-treated high-fat-fed mice. In agreement, pancreatic islet and beta cell areas were increased in (DAla^2^)GIP/xenin-8-Gln-treated mice, in line with the proliferative and anti-apoptotic beta cell actions of (DAla^2^)GIP/xenin-8-Gln, as has previously been reported for GIP [39, 40]. Consistent with our observations, the positive actions of GIP on islet and beta cell architecture have previously been shown to improve beta cell function and glycaemic control in animal models of type 2 diabetes [41]. Interestingly, there is a recent suggestion that xenin may inhibit GLP-1 secretion under certain circumstances [42]. However, such observations still need to be fully confirmed and the current findings would not indicate any obvious detrimental effects linked to reduced GLP-1 secretion or action by (DAla^2^)GIP/xenin-8-Gln. Moreover, GIP is known to stimulate GLP-1 secretion from the gut [43] as well as pancreatic alpha cells [44], affording GIP/xenin hybrids a clear advantage over xenin monotherapies. In keeping with improved beta cell dynamics in (DAla^2^)GIP/xenin-8-Gln-treated mice, the insulin-secretory and subsequent glucose-lowering effects of native GIP were substantially enhanced in these mice. As expected, the biological action of GIP was severely perturbed in high-fat-fed mice, akin to the situation in patients with type 2 diabetes [4]. It would be more than reasonable to link a major part of the positive therapeutic effects of (DAla^2^)GIP/xenin-8-Gln directly to augmentation of GIP action, as previously observed with other xenin-related compounds [10, 38]. Thus, the main rationale for inclusion of the xenin component of our novel hybrid peptide was essentially to enhance GIP-mediated actions [10, 38]. However, normalisation of blood glucose levels, as clearly demonstrated in both (DAla^2^)GIP- and (DAla^2^)GIP/xenin-8-Gln-treated mice, has also been shown to independently restore GIP insulin-secretory function in type 2 diabetes [5, 6]. In addition, we have already confirmed that xenin has significant glucose-lowering activity independent of the GIP receptor [38]. Therefore, further studies are required to systematically assess the direct and indirect beneficial effects of (DAla^2^)GIP/xenin-8-Gln in restoring GIP action in type 2 diabetes.

Interestingly, both activation and inhibition of GIP receptors has been postulated as a possible treatment option for type 2 diabetes and obesity [45]. Thus, GIP receptor agonism augments beta cell-induced glucose-dependent insulin secretion, akin to the actions of clinically approved GLP-1 mimetics [3]. This approach is very encouraging given that the issue of GIP sensitivity in type 2 diabetes is now being addressed. However, GIP is also believed to play a role in lipid metabolism and fat deposition [45]. Accordingly, GIP receptor antagonism can potentially protect against or even reverse many of the obesity-associated abnormalities of type 2 diabetes [46–48]. Similar to the conundrum regarding the clinical use of GIP receptor agonists or antagonists, both genetic knockout [49] and overexpression [50] of GIP has been shown to protect against obesity and development of diabetes. Taken together, this likely reflects the plasticity of cellular pathways linked to the regulation of energy balance. Nonetheless, it is clear from the current study that novel treatment options aimed at overcoming GIP resistance in type 2 diabetes have therapeutic potential.

In conclusion, the present study has demonstrated that the novel hybrid peptide analogue (DAla^2^)GIP/xenin-8-Gln has an impressive profile of beneficial metabolic effects in high-fat-fed mice, including improvement of glucose tolerance, insulin resistance and pancreatic islet morphology. Importantly, there was a clear augmentation of the biological action of native GIP in high-fat-fed mice, suggestive of restored GIP effectiveness by (DAla^2^)GIP/xenin-8-Gln. Further studies are required to fully evaluate the molecular mechanisms and full range of actions of (DAla^2^)GIP/xenin-8-Gln. However, it is evident that this stable GIP/xenin hybrid may represent an attractive potential new therapeutic for type 2 diabetes.

## Electronic supplementary material

Below is the link to the electronic supplementary material.

ESM 1 (PDF 95 kb)

## Abbreviations

DPP-4: Dipeptidyl peptidase 4
GIP: Glucose-dependent insulinotropic polypeptide
GLP-1: Glucagon-like peptide 1
Pal: Palmitate
