# Supplementary material for: An enzymatically stable GIP/xenin hybrid peptide restores GIP sensitivity, enhances beta cell function and improves glucose homeostasis in high-fat-fed mice
Source: Diabetologia. 2016 Dec 21;60(3):541–52. doi: 10.1007/s00125-016-4186-y (PMC6518372; doi:10.1007/s00125-016-4186-y)
Supplement: Supplementary file 1 — (PDF 95 kb) [file 125_2016_4186_MOESM1_ESM.pdf]

ESM methods

In vitro insulin release studies Cells were seeded at a density of 150,000 cells/well in 24-well plates (Nunc, Roskilde, Denmark) and allowed to attach overnight at 37°C. Culture medium was removed and cells were pre-incubated in Krebs–Ringer bicarbonate buffer (containing 0.5% (w/v) BSA, pH 7.4) supplemented with 1.1 mmol/l glucose for 40 min at 37°C. Following the pre-incubation, experiments (n=8) were performed in presence of glucose (5.6 or 16.7 mmol/l) with a range of concentrations of test peptides (10^{-12} to 10^{-6} mol/l) for 20 min at 37°C. After test incubations, aliquots of assay buffer were collected from each well and stored at -20°C prior to measurement of insulin.

Animals Acute animal studies were conducted in male NIH Swiss mice (12-14 weeks old, Harlan Ltd, UK) maintained on a standard rodent maintenance diet (10% fat, 30% protein and 60% carbohydrate, Trouw Nutrition, UK). Prior to commencement of longer term studies, all animals, except lean controls that continued on rodent maintenance diet, were maintained on a high-fat diet (45% fat, 35% carbohydrate and 20% protein, Special Diet Services, UK) for 12 weeks. This diet resulted in progressive body weight gain and hyperglycaemia. All animals were housed individually in an air-conditioned room at 22 ± 2 °C with a 12 h light:12 h dark cycle and had free access to food and water. Animal experiments were carried out in accordance with the UK Animals (Scientific Procedures) Act 1986.

Acute in vivo effects in lean mice For food intake studies, fasted (18 h) mice were given intraperitoneal (i.p) injections of peptides at a dose of 25, 100 or 250 nmol/kg bw. Mice were then allowed free access to normal chow for 180 mins and cumulative food intake measured. For glucose homeostasis and

insulin secretory studies, blood glucose and plasma insulin were measured immediately prior to and 15, 30, 60 and 105 min after i.p. administration of glucose alone (18 mmol/kg bw) or in combination with test peptides (each at 25 nmol/kg bw) in non-fasted mice.

Immunohistochemistry Tissue sections were deparaffinised, rehydrated and probed with primary antibodies: mouse anti-insulin antibody (1:500; Abcam, ab6995), guinea-pig anti-glucagon antibody (PCA2/4, 1:400; raised in-house) or rabbit anti-Ki67 (1:200; Abcam ab16667), as appropriate. We confirmed the specificity of all antibodies in blocking experiments using the native peptide. Sections were then incubated with secondary antibody, Alexa Fluor 488 goat anti-guinea pig IgG (1:400), Alexa Fluor 594 goat anti-mouse IgG (1:400) or Alexa Fluor 594 goat anti-rabbit IgG (1:400); respectively. TUNEL staining was performed using fluorescein *in situ* cell detection kit (Roche Diagnostics, Germany). The slides were viewed under a FITC (488 nm) or TRITC filter (594 nm) using a fluorescent microscope (Olympus system microscope, model BX51) and photographed using a DP70 camera adapter system. Islet parameters were analysed using Cell^{F} image analysis software (Olympus Soft Imaging Solutions, GmbH).

ESM Results

ESM Table 1 Amino acid sequence of xenin, xenin-8, xenin-8-Gln, (DAla^2)GIP and (DAla^2)GIP/xenin-8-Gln hybrid

Peptide Name	Amino acid sequence
Xenin	H-MET-LEU-THR-LYS-PHE-GLU-THR-LYS-SER-ALA-ARG-VAL-LYS-GLY-LEU-SER-PHE-HIS-PRO-LYS-ARG-PRO-TRP-ILE-LEU-OH
Xenin-8	H-HIS-PRO-LYS-ARG-PRO-TRP-ILE-LEU-OH
Xenin-8-Gln	H--**HIS-PRO-GLN-GLN-PRO-TRP-ILE-LEU-OH**
(DAla^2)GIP	**H-TYR-(d)ALA-GLU-GLY-THR-PHE-ILE-SER-ASP-TYR-SER-ILE-ALA-MET**-ASP-LYS-ILE-HIS-GLN-GLN-ASP-PHE-VAL-ASN-TRP-LEU-LEU-ALA-GLN-LYS-GLY-LYS-LYS-ASN-ASP-TRP-LYS-HIS-ASN-ILE-THR-GLN-OH
(DAla^2)GIP/xenin-8-Gln hybrid	**H-TYR-(d)ALA-GLU-GLY-THR-PHE-ILE-SER-ASP-TYR-SER-ILE-ALA-MET-HIS-PRO-GLN-GLN-PRO-TRP-ILE-LEU-OH**

Shared amino acid sequences in (DAla^2)GIP/xenin-8-Gln hybrid, and parent peptides, are shown in **bold** text.

ESM Table 2 Theoretical and experimental molecular masses and DPP-4 half-lives of test peptides

Peptide	Theoretical molecular mass (Da)	Experimental molecular mass (Da)	*In vitro* DPP-4 half-life (h)
Native GIP	4982.4	4986.1	<1
(DAla^2)GIP	4982.4	4982.4	>12
Xenin-25	2971.5	2970.8	-
Xenin-8	1046.3	1046.1	-
Xenin-8-Gln	1018.2	1018.9	-
(DAla^2)GIP/ xenin-8-Gln	2567.9	2565.3	>12

For calculation of experimental molecular masses, peptide samples (1 µg) were mixed with α-cyano-4-hydroxycinnamic acid (1 µg) and applied to a Voyager-DE BioSpectrometry Workstation and mass-to-charge (m/z) ratio verses peak intensity recorded by MALDI-TOF mass spectrometry. DPP-4 half-life was assessed by incubating peptides (37°C in 50 mmol/L TEA-HCl; pH 7.8) with 5 mU purified porcine DPP-4 for 0, 1, 2, 4, 8 and 12 h. Degradation profiles were obtained using rp-HPLC analysis and HPLC peak area data used to calculate percentage intact peptide remaining at each time point.
